# Maize Growth Promotion by Inoculation with an Engineered Ammonium-Excreting Strain of Nitrogen-Fixing *Pseudomonas* *stutzeri*

**DOI:** 10.3390/microorganisms10101986

**Published:** 2022-10-07

**Authors:** Shanshan Jiang, Jiang Li, Qingyu Wang, Changyan Yin, Yuhua Zhan, Yongliang Yan, Min Lin, Xiubin Ke

**Affiliations:** 1 Biotechnology Research Institute, Chinese Academy of Agricultural Sciences, Beijing 100081, China; 2 Key Laboratory of Development and Application of Rural Renewable Energy, Biogas Institute of Ministry of Agriculture and Rural Affairs, Chengdu 610041, China

**Keywords:** biological nitrogen fixation, *Pseudomonas*, ammonium excretion, ^15^N-dilution method, plant growth promotion

## Abstract

Diazotroph mutants designed using metabolic engineering to excrete surplus ammonium were used to enhance nitrogen fixation and plant growth, as the levels of nitrogen fixation attained with diazotrophs are insufficient for the plant’s needs. In this study, wild-type (A1501) and engineered ammonium-excreting (1568/pVA3) strains of nitrogen-fixing *Pseudomonas stutzeri* strains were tested in vitro based on plant growth-promoting traits, such as phosphate solubilization ability, indole acetic acid (IAA) production and nitrogenase activities, as well as ammonium excretion as affected by mannitol-mediated osmotic stress. The maize plant growth-promoting effect of the A1501 and 1568/pVA3 strains was evaluated in pots and in the field, and the ^15^N-dilution technique was employed to assess the proportion of plant nitrogen derived from nitrogen fixation. The results demonstrate that the 1568/pVA3 strain displayed higher IAA production and nitrogenase activity than A1501 and released significant quantities of ammonium. After 50 days, in all of the conditions assayed, maize inoculated with 1568/pVA3 accumulated more plant biomass (3.3% on average) and fixed N (39.4% on average) than plants inoculated with A1501. In the field experiment, the grain yield of maize was enhanced by 5.6% or 5.9% due to the inoculation of seeds with 1568/pVA3 in the absence or presence of exogenous N fertilizer, respectively. Therefore, the engineered *P. stutzeri* strain tested in the greenhouse and field was shown to perform better than the wild-type strain with respect to maize growth parameters and biologically fixed nitrogen.

## 1. Introduction

The inoculation of cereal crops with plant growth-promoting bacteria (PGPB) has been proven to be a sustainable way of increasing crop yields by facilitating plant growth through direct or indirect mechanisms [1,2]. In the selected cases, most PGPB can promote plant growth through multiple mechanisms, such as biological nitrogen fixation (BNF), phosphate solubilization, and indole-3-acetic acid (IAA) and siderophore production [3,4]. Moreover, PGPB can help plants withstand adverse conditions (e.g., drought/salinity) through the production of phytohormones and ACC deaminase [5]. Diazotrophic bacteria, such as *Azospirillum*, *Herbaspirillum*, and *Pseudomonas.* sp, belong to the plant growth-promoting rhizobacteria (PGPR) due to their ability to convert N_2_ into ammonia, and they have been used as inoculants in practice to improve plant biomass and nitrogen (N) content [6,7,8,9]. Nitrogen fixation is quantified using the ^15^N-dilution method, which compares the performance of nitrogen-fixing plants and non-fixing reference plants. This method has been used to obtain quantitative estimates of the proportion of plant N obtained from biological nitrogen fixation (BNF) [10]. Whether BNF from associative interactions contributes to plant growth promotion remains controversial. There is a positive response of plants to diazotrophic inoculation in some cases, where a substantial proportion of the nitrogen requirements of the plant can be provided by BNF under field conditions [7,11,12,13], while in other cases, there is not [9,14,15].

The nitrogen fixation efficacy seems to highly depend on inoculants, plant phenotypes, plant growing stage and environmental factors [7,16,17]. On the other hand, distinct from symbiotic associations, the transfer of fixed N from non-legume plant-associated diazotrophs to their hosts is considered a key problem [18]. This is attributed to associative, or free-living diazotrophs that fix dinitrogen sufficient for their own needs, and do not excrete N into the environment [19]. Nevertheless, the BNF activity of diazotrophic bacteria can potentially provide more N nutrition for the host plant, particularly if the diazotrophic bacteria are engineered to excrete ammonium [8,13,14,20,21]. It has been well documented that ammonium-excreting nitrogen-fixing bacteria can be obtained by means of two main strategies, including the inhibition of ammonium assimilation and interference with the mechanisms by which ammonium inhibits either nitrogenase synthesis or activity [22].

The root-associated bacterium *Pseudomonas stutzeri* A1501 was originally isolated from rice paddy soils [23,24]. Genomic sequencing led to the identification of a genomic island containing the *nif* genes as well as the rhizosphere competence traits required in the interaction with host plants [25]. This strain has been reported as being an endophyte that can promote rice plant growth through multiple mechanisms, such as providing rice plants with fixed N or producing 1-aminocyclopropane-1-carboxylate (ACC) deaminase [9,26,27,28]. Our previous study also showed that inoculation with *P. stutzeri* A1501 could increase maize plant growth and provide a significant amount of biologically fixed N to the host [29]. This indicates that strain A1501 can be a favorable agent for plant growth promotion testing because this bacterium can survive in the rhizosphere of cereal crops, compete against the indigenous soil microbiome and adapt to environmental stresses. In addition, genetic modifications on *P. stutzeri* such as gene deletion and recombination, can be simply obtained. For example, a recent study by Fox et al. [8] described the beneficial rhizobacterium *Pseudomonas protegens* Pf-5, containing the nitrogen fixation island from *P. stutzeri* A1501 via the recombinant cosmid X940, which showed high nitrogenase activity and ammonium production [20].

An ammonium-excreting strain of *P. stutzeri* A1501 has been obtained previously by constructing a mutant with the deletion of both ammonium transporter genes (*amtB*) and carrying a plasmid that expressed *nifA* from a constitutive promoter under nitrogen fixation conditions [30]. However, evidence of plant growth promotion by this recombinantly engineered strain is still lacking. Moreover, the response of such plant–microbe associations to environmental stresses (e.g., drought conditions) remains elusive. Therefore, in the present work, PGP traits of the *P. stutzeri* wild-type A1501 and excretion-ammonium (1568/pVA3) strains were characterized, and these bacterial inoculants were used to evaluate the growth promotion in maize plants under two water regimes (water stress and well-watered) in the greenhouse and the crop yields in fertilized field conditions.

## 2. Materials and Methods

### 2.1. Bacterial Strains and Media

The wild-type *Pseudomonas stutzeri* A1501 [31] and two mutant derivatives, including the engineered ammonium-excreting strain 1568/pVA3 and *nifH* mutant strain 1502, were tested in this study. The 1502 and 1568/pVA3 strains were constructed and described previously [30,32]. Briefly, the 1502 *nifH* mutant strain was constructed by means of the insertion of a Km cassette into the *Bgl*II site within the *nifH* coding sequence from the plasmid pUC4H. The *amtB* deletion mutant strain 1568/pVA3 was constructed by means of the replacement of the regions of ammonium transporter amtB1 and amtB2 with the chloramphenicol resistance gene *cat* (Cm); then, the pVA3 plasmid containing the *nifA* gene under the control of the constitutive Km promoter was introduced [30]. PCR and sequencing confirmed correct insertion. In both cases, wild strain and mutant derivatives were grown in LB or minimal lactate medium (medium K) at 30 °C [32]. Antibiotics were used at the following concentrations: 100 µg/mL ampicillin (Amp), 50 µg/mL kanamycin (Km), 10 µg/mL tetracycline (Tc), and 34 µg/mL spectinomycin (Spc). Osmotic stress was applied using 200 mmol/L mannitol, an osmotic component used generally to generate water deficit stress when added to the nutrient solution.

### 2.2. In Vitro Screening for Plant Growth-Promoting Traits

The wild-type and mutant derivatives used in this study were screened for a wide array of plant growth-promoting traits under normal buffered and osmotic stress (200 mmol/L mannitol) conditions. Indole acetic acid (IAA) production was tested using Salkowski’s reagent [33]. Phosphate solubilization was determined in Pikovskaya’s agar medium, according to Fiske and Subbarow [34]. The strains that formed halos around the colonies were considered positive. The phosphate-solubilizing ability of bacteria was measured as the phosphate solubilization index (PSI) and was measured using the following formula [35].

PSI = (Colony diameter + Halo zone diameter)/Colony diameter

To determine the amount of ACC deaminase (1-aminocyclopropane-1-carboxylate deaminase) activity, the amount of aketobutyrate generated from the cleavage of ACC was measured [36]. The ACC deaminase activity was expressed as the amount of a-ketobutyrate produced per milligram of protein per hour. All tests were replicated three times.

### 2.3. Nitrogenase Activity Assays

The nitrogenase activity was measured following the acetylene reduction assay [37] as follows. Cells from an overnight culture in LB medium were centrifuged and resuspended in a 60 mL flask containing 10 mL of N-free medium K at an OD_600_ of 0.1. The suspension was incubated at 30 °C, with vigorous shaking, under an argon atmosphere containing 0.5% oxygen and 10% acetylene. Gas samples (0.25 mL) were taken at regular intervals to determine the amount of ethylene produced. Samples were analyzed on a polydivinylbenzene porous bead GDX-502 column using a gas chromatograph SP-2100 fitted with a flame ionization detector (Beijing Beifen-Ruili Analytical Instrument Co. Ltd., Beijing, China). The ethylene content in the gas samples was determined with reference to an ethylene standard. Each experiment was repeated three times.

### 2.4. Ammonium Excretion Determination

To determine the excretion rate of ammonium, cells from an overnight culture in the LB medium were centrifuged and resuspended in a 60 mL flask containing 10 mL of N-free medium K at an OD_600_ of 0.1. The cells were incubated in an N_2_ atmosphere containing 0.5% oxygen. The concentration of ammonium in the supernatant was determined fluorometrically by means of microscale analysis [38] after 72 h. Each experiment was performed three times.

### 2.5. Pot Experiments and Inoculation Method

The experiments were performed in a greenhouse with ambient day/night cycles and temperatures ranging between 20 and 30 °C at the CAAS (Beijing, China). The soil was collected from farmland and was well homogenized, then air-dried, sieved (2 mm mesh size) and stored at room temperature. The soil characteristics were described previously [29]. Before use, the soil was sterilized by means of γ-irradiation (60 kGy; ^60^Co; 72 h), and the sterilization efficiency was tested through plate counting. Three kilograms of soil were added to each polyethylene pot and amended with KCl and Na_2_HPO_4_ at amounts of 17 mg of K and 50 mg of P per kilogram of soil, respectively. Two water regimes were set up to simulate water stress and well-watered conditions: 20% and 60% moisture were achieved by adding appropriate volumes of sterile water and mixing thoroughly in the pots. The soil moisture was monitored by a water content detector (Meacon, China) and remained stable during the maize growth.

Maize (*Zea mays* L, genotype hybrid Zhengdan 958) seeds were surface-sterilized with 2% NaClO for 1 min followed by 70% ethanol for 10 min, and rinsed with sterile water three times. After sterilization, seeds underwent accelerating germination in a climate chamber at 25 °C (16 h light/8 h dark cycle). After germination, uniform seedlings were selected for continued growth in the greenhouse. *P. stutzeri* A1501 and mutant derivatives were grown in LB medium to an OD_620_ = 1.0 (approx. 8.8 × 10^8^ cells mL^−1^). After centrifugation at 5000 rpm for 30 min, the supernatant was discarded and the cells were resuspended with the same volume of sterile NaCl solution (0.8%). Inoculation was performed by dipping the maize seedlings’ roots in bacterial suspensions overnight before transplanting them to the soil. For each set, four inoculation conditions were used with 16 repetitions per condition: (i) inoculation with *P. stutzeri* A1501, (ii) inoculation with *nifH* mutant 1502, (iii) inoculation with ammonium-excreting strain 1568/pVA3, and (iv) inoculation with a sterilized suspension of A1501. After 50 days of growth, the maize plants were harvested to determine the weight and length of the shoot/root. The length of the root was obtained by measuring the longest root. Rhizospheric soil, defined as the soil closely adhering to the roots after hand-shaking, was collected to measure organic C, ammonium and nitrate concentrations.

### 2.6. ^15^N Dilution Assay

Soil microcosms planted with maize were treated with approximately 50 mg/kg soil ^15^N-ammonium sulfate (10.18% atom, Shanghai Research Institute of Chemical Industry, China), which was added in advance and mixed well. After 50 days, plant material was sampled for ^15^N measurements, including foliage N concentration and atom% ^15^N excess by mass spectrometry at the Institute of Environment and Sustainable Development in Agriculture, CAAS (Beijing). The changes in atom% ^15^N excess were determined and used to quantify the amount of fixed N. The percentage of N derived from atmospheric N_2_ (% Ndfa) via BNF for individual maize plants was calculated by using the equation:

% Ndfa = (1 − % ^15^N_F_/% ^15^N_NF_) × 100

where percent ^15^N is the average per milligram of ^15^N excess in the fixing (F) and non-fixing (NF) maize, respectively. The uninoculated maize plant was used as the reference for the calculation of %Ndfa, assuming that this plant was not fixing N or had insignificant nitrogen fixation.

Total N fixed (kg/plant) was calculated by the equation:

N_fixed_ = N_t_ × % Ndfa × Biomass

where N_t_ is total N concentration. Biomass (kg/plant) was determined by means of destructive sampling of each plant for both above- and below-ground dry mass.

### 2.7. Field Experiment for Crop Yield

Field trials were carried out from 10 January to 30 April 2021 at an experimental field station (108°47′9.28″ E, 18°36′17.25″ N) in Ledong, Hainan province, China. The climate is classified as a tropical maritime monsoon climate. In the 2021 trial, the minimum and maximum temperatures were 20.1 °C in January and 32.4 °C in April, respectively. The average precipitation was 25 mm during spring. The properties of soil were described as follows: pH, 6.7; NH_4_^+^ -N: 1.74 mg/kg; NO_3_^−^ -N, 3.41 mg/kg; total N: 0.17%; and organic C: 8.87 mg/kg. A completely randomized block design was implemented, and three replicate plots were used for each treatment. Each plot was 5.0 m (wide) by 8.0 m (long), and the next plot was 1.0 m away in all directions. Three days before inoculation, seeds were treated with insecticides (imidacloprid, 30.9%; tebuconazole, 1.1%) according to the technical recommendations for the crop in China. Inoculants were prepared as described above. Approximately 2 kg seeds were soaked in a 50 mL bacterial suspension (~2 × 10^9^ cells/mL) for 4 h and air-dried at 25 °C before manual sowing. At least 100 maize seeds were sowed in five rows per plot. Nitrogen treatments included three levels of N (urea) application: 0, 75 and 100 kg of N per hectare. Half of the total N was applied as the basic fertilizer, and the remaining urea was applied at successive intervals of 40 days. Potassium sulfate was applied at 45 kg of K per hectare, and superphosphate was applied at 75 kg of P per hectare prior to sowing. Herbicides were used in all treatments according to the technical recommendations. Five irrigations were applied during the whole plant growing season. The maize crop was harvested about 100 days after sowing. Twenty plants per plot were randomly collected for the measurements of biometric parameters: shoot height, shoot dry weight (including biomass of cobs/grains), ear height, length and width of leaf, the row numbers per ear and grain numbers per row, and crop yield.

### 2.8. Root Sampling and Bacterial Counts

In order to test for the presence of bacterial inoculants in roots during plant growth, the populations of bacteria attached to the root surface were estimated by the most probable number (MPN) method. After sampling, roots were weighted and washed three times in a sterile PBS solution. Serial dilutions (10^−5^–10^−8^) of each sample were plated on K medium supplemented with antibiotics and incubated at 30 °C for 18–20 h prior to obtaining a colony-forming unit (CFU) count. It should be mentioned that the MPN method only confirmed the total bacterial population in the rhizosphere or roots instead of accurately targeting the wild-type *Pseudomonas stutzeri* A1501 or two mutant derivatives. Despite the limitations of the MPN method, our aim here was to confirm if the wild-type and mutant derivatives could colonize tissues at a similar rate and establish themselves in the rhizosphere. To avoid inaccurate estimation of inoculants, high-resolution methods such as the PCR technique with target-specific primers could be considered in the future.

### 2.9. Statistical Analysis

Spearman’s correlation analyses were performed to assess the relationships among datasets. For multiple comparisons, one-way ANOVA with Tukey’s post-hoc tests (α = 0.05) was performed using the SPSS 19.0 package (IBM SPSS). All differences at *p* < 0.05 were reported as significant.

## 3. Results

### 3.1. Properties of the Wild-Type and Mutant Derivatives

**Figure 1:**
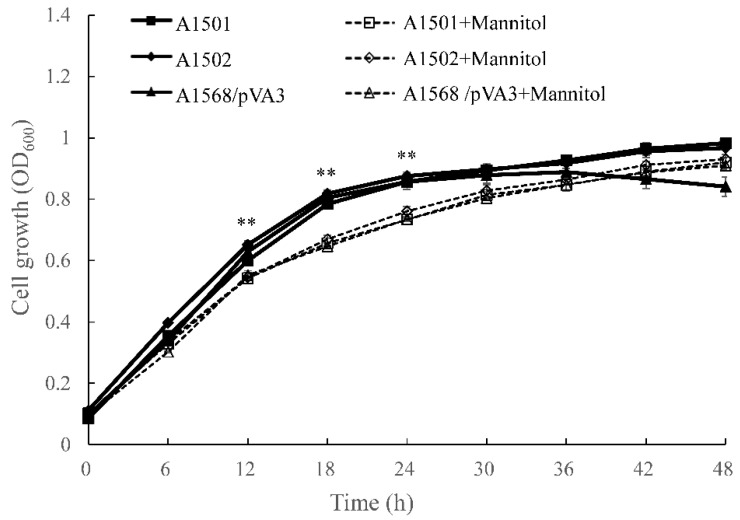
Influence of mannitol on the bacterial growth rates of wild-type *P. stutzeri* A1501 and two mutant derivatives, including the ammonium-excreting strain 1568/pVA3 and the *nifH*-mutant 1502. Data are means ± SE (*n* = 3). Different letters above the boxes indicate a significant difference (*p* < 0.05). The statistical analysis was carried out with ANOVA followed by the Duncan test. ** *p* value ≤ 0.01. All values are means ± SE (n = 3).

The growth properties of the wild-type A1501 strain, the ammonium-excreting strain 1568/pVA3 and the *nifH*-mutant 1502 as affected by mannitol-mediated osmotic stress were tested in medium K. As shown in Figure 1, the growth of the three strains was significantly inhibited when they were exposed to mannitol. Without mannitol, the A1501, 1568/pVA3 and 1502 strains all showed exponential growth from inoculation until the stationary phase was reached after approximately 24 h. However, tor the mannitol treatment, growth was inhibited initially in some cases, and there was a long lag phase (~18 h) before growth was observed (Figure 1).

After the inoculation of wild-type A1501 and the two mutant derivative strains on Pikovskaya’s medium plates for five days, all the strains generated the expected halo zones and showed similar phosphate solubilization indexes (PSI) (Table 1). IAA production was recorded after 24 h of incubation in all of the strains. In the presence of mannitol, the efficiency of IAA synthesis was significantly enhanced. The strain 1568/pVA3 produced the highest amount of IAA in all the treatments (Table 1). After 24 h of incubation, the strains showed little variation in the ACC deaminase activity, ranging from 3.74 to 3.88 μmol a-ketobutyrate mg protein^−1^ h^−1^ (Table 1), and no significant effect of mannitol treatment was observed.

The nitrogenase activity of wild-type A1501 and the two mutant derivatives as affected by mannitol-mediated osmotic stress was determined under diazotrophic conditions. The nitrogenase activity for A1501 (1618.2 nmol of ethylene mg protein^−1^ h^−1^) was similar to that of 1568/pVA3 (1792.3 nmol of ethylene mg protein^−1^ h^−1^) (Table 1). Meanwhile, nitrogenase activity decreased by 3.4% for A1501 (1564.8 nmol of ethylene mg protein^−1^ h^−1^) and 7.0% for 1568/pVA3 (1675.1 nmol of ethylene mg protein^−1^ h^−1^) in the presence of mannitol. As expected, the *nifH* mutant strain 1502 failed to fix nitrogen in all treatments (Table 1).

The ammonium excretion capacity of the strain 1568/pVA3 was indirectly determined by measuring the NH_4_^+^ concentration in a free nitrogen medium. After 72 h of incubation, the total concentration of ammonium excreted by mutant 1568/pVA3 reached 20.3 μM as compared to 18.2 μM when treated with mannitol (Table 1).

### 3.2. Colonization of Wild-Type and Mutant Strains on the Maize Root Surface

The extent of the colonization of root tissue was evaluated by means of the most probable number (MPN) method under greenhouse conditions. Fifty days after inoculation, significantly increased bacterial root colonization was observed, in comparison to plants from the non-inoculated controls (Table 2). However, there were no significant differences in CFUs from the root samples between plants inoculated with mutants or with the wild type.

### 3.3. Effects of Wild and Mutant Strains Inoculation on Maize Growth Promotion

**Figure 2:**
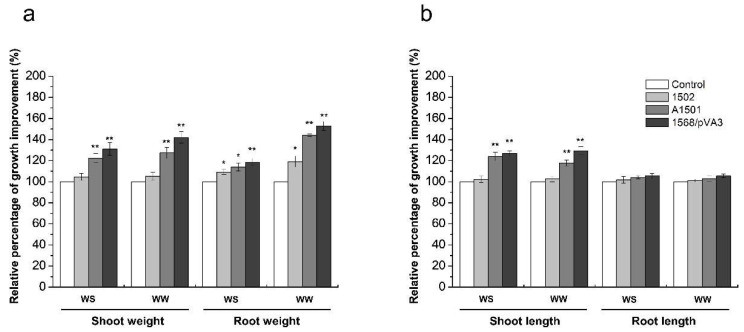
Relative percentage of growth improvement for the weight of shoots and roots (**a**) and the length of shoots and roots (**b**) induced by *P. stutzeri* inoculation in maize plants. Control, plant without inoculation; A1501, plant inoculated with wild-type; 1568/pVA3, ammonium-excreting strain; 1502, *nifH*-mutant. WS—water stress condition; WW—well-watered condition. The values are mean ± standard deviation (n = 16). The statistical analysis was carried out with ANOVA followed by the Duncan test. * *p*-value ≤ 0.05 or ** *p*-value ≤ 0.01.

To test whether the ammonium-excreting strain 1568/pVA3 could stimulate plant growth under water stress or well-watered conditions, the increases in root and plant weight and stem and root length were measured in inoculated maize plants and compared with the plants inoculated with wild-type A1501, *nifH* mutant 1502 and uninoculated controls. After 50 days of growth, the maize plant production was significantly affected by the water treatments (Table 1). Greater weight and length of shoots and roots as indexes of maize growth were always detected under well-watered conditions. Inoculation of maize with 1568/pVA3 significantly increased shoot weight by 31.2 and 42.0% as compared to the controls under water stress and well-watered conditions (*p* < 0.01), respectively (Figure 2a). Similarly, A1501 produced significant (*p* < 0.01) increases of 22.5% and 27.3% when compared to the uninoculated plants under water stress and well-watered conditions (*p* < 0.01), respectively (Figure 2a), while plants inoculated with only 1502 demonstrated an increase in shoot weight with respect to the uninoculated plants (Table 2 and Figure 2a). Additionally, maize plants treated with the 1568/pVA3 and A1501 strains presented longer shoots (*p* < 0.01) than the controls without inoculation under the two water regime conditions, respectively (Table 2, Figure 2b). For the roots, root weight significantly increased with all of the inoculation treatments with the 1568/pVA3, A1501 and 1502 strains (Table 2 and Figure 2a) relative to the control without inoculation under the two water regime conditions, respectively. However, the treatments using only 1568/pVA3 and A1501 generated a slight increase (4.3% on average) in root length compared to the controls (Figure 2b).

### 3.4. Effects of Inoculation with P. stutzeri Strains on Maize N Concentrations

The impact of the inoculation of *P. stutzeri* wild-type A1501 and ammonium-excreting strain 1568/pVA3 on the nitrogen concentrations of maize plants was evaluated using *nifH*-mutant 1502 as a control inoculum in pot experiments under water stress and well-watered conditions, respectively. The ^15^N abundance, δ^15^N and nitrogen concentrations due to inoculation with 1568/pVA3 and A1501 showed significant differences compared with the controls (Table 3). Fifty days after inoculation, a significantly lower δ^15^N value was noted in the aerial parts of 1568/pVA3- and A1501-treated plants than those of *nifH* mutant-treated plants, suggesting that BNF was active during the growing period. For the 1568/pVA3-inoculated plants, the percentage of N derived from the atmosphere (%Ndfa) in the aerial parts of maize averaged 18.9% under water stress conditions as compared to 28.4% under well-watered conditions (Table 3). Correspondingly, individual maize plants could obtain 0.13 g of N and 0.39 g of N from BNF under water stress and well-watered conditions, respectively. For the A1501-inoculated plants, the mean %Ndfa was approximately 15.1% and 23.3% under water stress and well-watered conditions (Table 3), respectively. Accordingly, individual maize plants could obtain 0.09 g of N and 0.29 g of N from BNF under water stress and well-watered conditions, respectively.

The content of organic C, ammonium and nitrate nitrogen in the rhizospheric soil of the maize plants was determined at 50 d (Table 4). The rhizospheric soil from the maize plants inoculated with 1568/pVA3 contained significantly more NH_4_^+^ than the soil that received the A1501 and 1502 inoculation treatments and the non-inoculation control. The same was true for nitrate concentration. The organic C levels were not significantly different between treatments.

### 3.5. Crop Yield in Field Experiment

**Figure 3:**
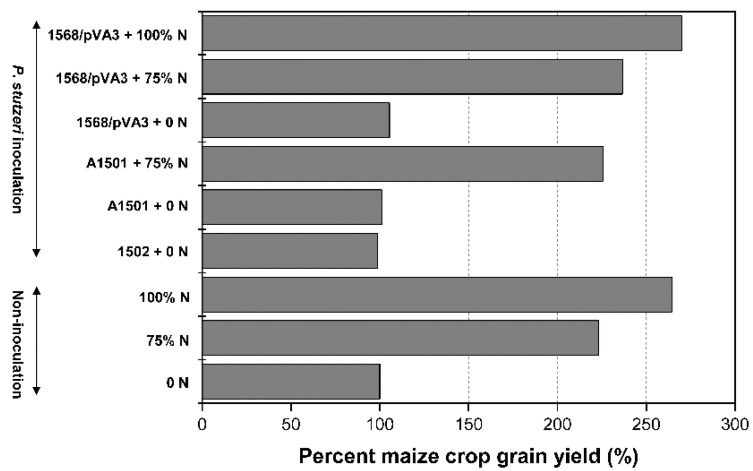
Effect of *P. stutzeri* inoculation on the grain yield of maize in the field experiment. 0 N, 0 kg of N ha^−1^, maize plants neither fertilized with urea nor inoculated with strains were considered as control (100%); 75% N, 75 kg of N ha^−1^; 100% N, 100 kg of N ha^−1^; 1502 + 0 N, 0 kg of N ha^−1^ plus inoculation with *nifH*-mutant 1502; A1501 + 0 N, 0 kg of N ha^−1^ plus inoculation with wild-type A1501; A1501 + 75% N, 75 kg of N ha^−1^ plus inoculation with wild-type A1501; 1568/pVA3 + 0 N, 0 kg of N ha^−1^ plus inoculation with ammonium-excreting strain 1568/pVA3; 1568/pVA3 + 75% N, 75 kg of N ha^−1^ plus inoculation with 1568/pVA3; 1568/pVA3 + 100% N, 100 kg of N ha^−1^ plus inoculation with 1568/pVA3. The values are mean ± standard deviation (n = 20).

As shown in Table 5 and Figure 3, multiple biometric parameters and grain yield of maize were determined after harvesting in the field experiment, indicating that the fertilization or inoculation treatments or inoculation associated with base fertilization induce obvious modifications to maize growth. Inoculation of seeds with *P. stutzeri* 1568/pVA3 without urea application (1568/pVA3 + 0 N) showed higher agronomic traits compared with the non-inoculated 0 N treatment (0 N), and significant differences were observed in shoot N concentrations (NC), width of leaf (WL), diameter of shoot (DS), thousand seed weight (TSW) and grain yield (GY).

Seeds inoculation with 1568/pVA3 significantly increased the yield of maize by 5.6% (0.5 kg per 20 m^2^) in the absence of any urea application (Figure 3). In the presence of 75% N (75 kg N ha^−1^) application, there was a 5.9% increase in grain yield when the seeds were inoculated with 1568/pVA3 in comparison with only 75% N. The inoculation with 1568/pVA3 associated with the base fertilization of 100 kg of N ha^−1^ (1568/pVA3 + 100% N) was statistically equal to the only 100% N control (Table 5), while no significant differences in grain yield were observed when seeds received the A1501 and 1502 inoculants.

## 4. Discussion

Our experiment was designed to verify whether an engineered ammonium-excreting strain of *P. stutzeri*, 1568/pVA3, could stimulate maize plant growth and provide more fixed N than the wild-type A1501 strain under different water regime conditions in a greenhouse trial. The potential of seeds inoculated with *P. stutzeri* regarding crop yield promotion and N fertilizer reduction was also tested in field conditions by comparing the agronomic traits and productivity of maize treated with inoculants and different doses of urea application. Furthermore, plant growth-promotion traits and the effectiveness of nitrogen fixation were compared between wild-type and ammonium-excreting strain in a batch culture system. 

It has been well established that ammonium excretion strain can be achieved through chemical mutagenesis and genetic manipulation [22]. Previous work has confirmed that *P. stutzeri* 1568/pVA3 is an *amtB* deletion strain carrying a plasmid containing *nifA*, and is able to fix nitrogen constitutively under culture conditions [30]. In this present study, equivalent growth rates were found among the wild-type A1501 strain, the *nifH* mutant strain 1502 and the ammonium-excreting strain 1568/pVA3 in two mannitol treatments during incubation (Figure 1). This implies that genetic modifications such as the deletion of *nifH* or *amtB* genes and recombination only affect nitrogen-fixing- or ammonium-excreting-related functions. Therefore, we assumed that the associations between the plant and the mutant or the wild-type strains, such as colonization, competition with indigenous microorganisms, and biofilm formation and tendency, showed no differences in response to the environmental factors. Indeed, the strains successfully colonized the plant roots, as shown in Table 2, and equal colonization rates of mutant and wild-type strains were determined by means of the re-isolation inoculants from the rhizoplane of maize and subsequent plate counting.

In this study, we reported that *P. stutzeri* 1568/pVA3 can excrete ammonium at up to 10 μM after 24 h of incubation [30] and 18–20 μM after 72 h of incubation. In parallel, in the greenhouse trial, the ammonium concentration was more than four times higher in the rhizospheric soil of maize inoculated with 1568/pVA3 compared with that inoculated with the A1501 wild-type, *nifH* mutant or non-inoculation (Table 4). Similarly, it was recently observed that inoculation with an ammonium-excreting strain was associated with the availability of nitrogen compounds in maize and wheat cultivation soil [20,39,40]. This result suggests that the beneficial effect of *P. stutzeri* 1568/pVA3 inoculation on plant growth promotion can be attributed to the production and excretion of a high amount of NH_4_^+^ into the environment. Obviously, in the greenhouse experiment, compared with wild-type-inoculated plants, the engineered 1568/pVA3 strain was shown upon inoculation to increase the shoot weight and length by at least 10% under the two regime conditions.

Meanwhile, it was interesting that the root weight (more than 50%) was also markedly stimulated by the 1568/pVA3 inoculation treatment compared with the uninoculated control (Figure 2). Although all tested inoculants showed significant stimulation of root growth, only the engineered ammonium-excreting inoculants had an additional benefit related to this adaptation due to the simultaneous increase in rhizosphere ammonium concentrations. In this light, a more detailed characterization of root growth effects (e.g., root hairs, root diameter, volume, etc.) would be needed in further study. This result matches previous research showing that inoculation with ammonium-excreting strains is associated with higher plant productivity. For instance, wheat plants inoculated with an *Azospirillum brasilense* ammonium-excreting strain showed up to 50% higher whole-plant dry weight than those inoculated with the wild-type strain [41]. Maize plants inoculated with recombinant *Pseudomonas protegens* Pf-5 X940 showed an increase of 115% in biomass accumulation with respect to plants inoculated with the non-N2-fixing control [8]. More recently, wheat plants inoculated with an ammonium-excreting strain of *Azospirillum brasilense* showed 30% and 31% higher shoot and root dry weight than those inoculated with the wild-type strain [21]. Thus, these results strongly support the hypothesis that diazotrophic PGPB mutants with alterations in ammonium excretion are more efficient at improving plant biomass than their respective wild-type strains.

Additionally, in the field trial, positive plant growth-promoting effects of inoculation with 1568/pVA3 were also observed. The 1568/pVA3 inoculation induced higher yield components, mainly evidenced by the increase in the diameter of shoots, the thousand seed weight, the grain yield, etc. The grain yield of maize was generally equal to that reported in other studies (around 5–9 t/ha) [42,43] and higher than the average maize yield (6.0 t/ha in 2020, data from Knoema Data Hub Catalog) in China. In addition, the inoculation of maize with 1568/pVA3 significantly promoted higher N concentrations (3.4–5.2%) in shoots than the 0 N treatment (*p* < 0.05). This result basically agrees with the observations in the greenhouse experiment, indicating a 2.2–6.0% increase in plant N concentrations in response to inoculation with 1568/pVA3 (Table 3). However, in comparison with the pots experiment, a lower expression of effects was observed in the field experiment. For example, in the field trial, inoculation of maize with 1568/pVA3 induced a slight increase (1.5%) in shoot biomass as compared to the controls, while more than a 40% increase was observed in the pots experiment under the same conditions. Furthermore, even with the supply of N fertilizers, only an approximately 6% improvement in grain yield (1568/pVA3 inoculation treatment vs. non-inoculation control) was achieved at the time of harvest. This indicates that greenhouse- and field-measured plant growth promotion was not fully correlated, which could be attributed to climate, soil types and chemistry, and the inoculation method. 

Greenhouse studies can more easily distinguish bacterial inoculation effects compared to field studies. Indeed, more stressful growing conditions (competition, disease, drought) may reduce the growth-promoting impact outside. In particular, the soil used in the pots experiment was sterilized, which may benefit root colonization by bacteria and plant growth by eliminating harmful factors such as pathogens and insects. Referring to the grain yield of maize, when seeds inoculation with 1568/pVA3 plus 75% of the supplemental dose of N, the grain yield was superior (~5.9% increase) to that of non-inoculated plants receiving 75% N, but accounted for 89.5% of that of plants receiving 100% N. Therefore, it was estimated that a saving of 25% for the N fertilizer was almost achieved with the inoculation of maize seeds with the engineered ammonium-excreting strain of *P. stutzeri* in this study. Reports from the literature showed that engineered *Azospirillum* and *Azotobacter* strains could reduce the consumption of N fertilizers in maize or wheat in a range of 25 to 130 g of N per hectare [39,42,43]. This high variation in plant N concentrations may rely on many factors, such as plant cultivars, soil type and nutrient conditions, climate differences, and the inoculation method, which could influence inoculant performance and plant development [42,43,44].

By using the ^15^N-dilution method, a significantly large fraction of the fixed N (18.9–28.4%) provided by *P. stutzeri* 1568/pVA3 inoculated in maize was taken up under the two water regimes, respectively. In addition, maize plants inoculated with 1568/pVA3 displayed an increase of 5.1 and 5.6% in the amount of fixed N compared with the non-inoculation control under water stress and well-watered conditions, respectively. This indicates that the changes in the ammonium-excreting characteristics of the *P. stutzeri* strain may relate to the high nitrogen fixation efficacy. This observation is consistent with those previously reported in a C4 plant *Setaria viridis* system, where inoculation with an ammonium-excreting strain of *Azospirillum brasilense* in *Setaria viridis* grass led to the incorporation of 16-fold more nitrogen than the wild-type strains [13]. Therefore, inoculation with 1568/pVA3 showed more beneficial effects on biomass accumulation and N uptake, which we tentatively link to the abilities to excrete ammonium and produce higher amounts of IAA. In this case, significantly higher IAA production of 1568/pVA3 was also observed, especially when treated with mannitol (Table 1). Previous studies have suggested that IAA can positively promote plant growth, such as lateral root development and root elongation [45]. Interestingly, it is noted that maize plants inoculated with the *nifH*-mutant strain 1502 displayed a significant increase of 14.2% in root weight on average, with respect to uninoculated plants. It is postulated that apart from BNF, the minor root growth stimulation may be attributed to other bacterial characteristics, such as the production of phytohormones [46]. Indeed, of the three strains, similar results regarding P solubilization ability and ACC deaminase activity were found among the wild-type and *nifH* mutant strains (Table 1).

In a previous study, we observed that more ammonium was excreted when a plasmid expressing constitutive levels of *nifA* was introduced into the *amtB* deletion strain [30]. The level of ammonium excretion of 1568/pVA3 in the medium was similar to that of an *Azotobacter vinelandii* mutant [47], which could produce 20 μM ammonium, but was much lower than that of an *Azospirillum brasilense* mutant [21] and recombinant *Pseudomonas* containing the X940 cosmid [20], which could excrete a concentration of 260–1000 μM NH_4_^+^. As reported earlier [21], a spontaneous single mutation in glutamine synthetase (GS) of *Azospirillum brasilense* HM053 lead to high ammonium excretion (up to 2 mM NH_4_^+^ after 72 h of incubation). It was suggested that HM053’s ability to excrete so high a concentration of ammonium was related to its low GS activity, resulting in a deficiency of NH_4_^+^ assimilation and explaining the excretion of excess ammonium produced during nitrogen fixation. For 1568/pVA3, this engineered strain was able to release ammonium after the deletion of both *amtB* genes, indicating that the ammonium transporter *amtB* was also involved in the regulation of nitrogenase activity [30]. The differentiation in the ammonium excretion abilities may be due to the regulation of nitrogen metabolism by different systems and metabolic engineering approaches. In addition, the stability of the plasmid pVA3 was not determined either in the greenhouse or the field experiment. It is possible that the plasmid pVA3 was lost from the 1568/pVA3 strain in the absence of selective pressure (e.g., absence of antibiotics), which may have led to a decrease in the rate of ammonium excretion. In combination with the fact that water stress leads to a significant decrease in the ammonium excretion ability of the strain 1568/pVA3, this thus demonstrates high potential for the metabolic engineering of *P. stutzeri* A1501, such as enhancing its ammonium-excreting capacity or conditional release of ammonium or stress tolerance and resilience according to the surrounding carbon and nitrogen status through synthetic biology approaches.

*Pseudomonas* has been found to be beneficial for cereal crops, such as maize, wheat and potato, and has been used as an inoculum in practice [48,49,50]. *P. stutzeri* is distributed widely in the environment, occupying diverse ecological niches, and can be considered an opportunistic pathogen in humans [51]. Thus, its environmental safety needs to be illustrated further.

In summary, our results show that an engineered ammonium-excreting strain of *P. stutzeri* 1568/pVA3 was able to release ammonium and demonstrated higher nitrogenase activity and IAA production than wild-type A1501. Significant inhibition of mannitol-induced osmotic stress on the wild-type and mutant derivatives was observed. Our results also demonstrate that the inoculation of maize with an ammonium-excreting strain of *P. stutzeri* led to significant plant growth promotion and supplied the plant with BNF-derived N under greenhouse conditions. Compared with the wild-type strain, the greater nitrogen fixation efficacy of the ammonium-excreting strain and its effectiveness as a PGPB were also confirmed under water stress and well-watered conditions. In the field experiment, the seeds’ inoculation with *P. stutzeri* 1568/pVA3 improved maize growth and grain yield and reduced N fertilizer demand. The evidence presented here emphasizes the application of engineered ammonium-excreting strain of *P. stutzeri* as an effective crop inoculant to enhance plant growth. Further analysis might be necessary to focus on improving competitiveness with native microbes and resistibility to abiotic stress by genetic manipulation.

## Figures and Tables

**Table 1:** Qualitative analysis of the phosphate solubilization ability, IAA production, ammonium excretion, and ACC deaminase and nitrogenase activity of the *P. stutzeri* A1501 wild-type strain, the ammonium-excreting strain 1568/pVA3, and the *nifH*-mutant 1502. n.d.—not detected. Different letters (a–c) indicate a significant difference (*p* < 0.05) according to ANOVA analysis.

Strains	Mannitol-Treated Condition	Phosphate Solubilization Ability			IAA Production (mg/L)	ACC Deaminase Activity (μmol a-Ketobutyrate mg Protein^−1^ h^−1^)	Nitrogenase Activity (nmol of Ethylene mg Protein^−1^ h^−1^)	Extracellular Ammonium Concentration (μM)
		Diameter of Halo (cm)	Diameter of Colony (cm)	PSI				
1568/pVA3	0	1.70 ± 0.01 a	1.11 ± 0.02 a	1.53 ± 0.04 a	28.61 ± 1.11 b	3.88 ± 0.10 a	1792.3 ± 98.7 b	20.3 ± 0.4 b
	200 mM	1.74 ± 0.02 a	1.13 ± 0.02 a	1.54 ± 0.06 a	32.67 ± 0.99 c	3.74 ± 0.06 a	1675.1 ± 112.9 ab	18.2 ± 0.2 a
A1501	0	1.68 ± 0.04 a	1.06 ± 0.03 a	1.59 ± 0.12 a	22.88 ± 1.17 a	3.80 ± 0.09 a	1618.2 ± 101.4 ab	n.d.
	200 mM	1.72 ± 0.02 a	1.10 ± 0.02 a	1.57 ± 0.07 a	29.26 ± 2.47 b	3.85 ± 0.11 a	1564.8 ± 90.6 a	n.d.
*nifH*-mutant	0	1.69 ± 0.03 a	1.12 ± 0.01 a	1.51 ± 0.05 a	24.79 ± 2.30 a	3.76 ± 0.05 a	n.d.	n.d.
	200 mM	1.73 ± 0.03 a	1.12 ± 0.04 a	1.55 ± 0.10 a	28.14 ± 1.03 b	3.81 ± 0.07 a	n.d.	n.d.

**Table 2:** Shoot weight (SW), root weight (RW), shoot length (SL), root length (RL) and most probable number (MPN) of the *P. stutzeri* A1501 wild-type strain or mutants on roots in a greenhouse experiment under two water regimes. The values are mean ± standard deviations (n = 16). Different letters (a–c) indicate a significant difference (*p* < 0.05) according to ANOVA analysis. WS—water stress condition; WW—well-watered condition.

Inoculant Treatments	SW (g)		RW (g)		SL (cm)		RL (cm)		Bacteria Root Colonization (CFU g^−1^)	
	WS	WW	WS	WW	WS	WW	WS	WW	WS	WW
1568/pVA3	22.7 ± 0.9 b	53.1 ± 2.8 c	5.1 ± 0.2 c	10.4 ± 0.9 c	61.1 ± 4.2 b	74.3 ± 2.8 b	21.2 ± 3.8 a	30.7 ± 3.5 a	8.15 × 10^5^ b	1.54 × 10^7^ b
A1501	21.2 ± 0.8 b	47.6 ± 3.9 b	4.9 ± 0.3 bc	9.8 ± 0.6 c	59.7 ± 3.6 b	67.6 ± 4.0 b	20.9 ± 2.4 a	29.8 ± 2.6 a	9.09 × 10^5^ b	1.46 × 10^7^ b
*nifH*-mutant	18.1 ± 1.0 a	39.4 ± 2.7 a	4.7 ± 0.1 b	8.1 ± 0.3 b	49.3 ± 3.0 a	58.9 ± 1.8 a	20.5 ± 3.9 a	29.4 ± 3.3 a	1.04 × 10^6^ b	1.38 × 10^7^ b
No inoculation	17.3 ± 0.5 a	37.4 ± 3.4 a	4.3 ± 0.1 a	6.8 ± 1.1 a	48.2 ± 1.4 a	57.4 ± 3.3 a	20.1 ± 4.2 a	29.1 ± 4.2 a	7.02 × 10^2^ a	2.26 × 10^2^ a

**Table 3:** Effects of the inoculation with *P. stutzeri* A1501 wild-type and mutant strains on nitrogen concentrations and atom % ^15^N excess of maize stems. The values are mean ± standard deviations (n = 20). Different letters (a–b) indicate a significant difference (*p* < 0.05) according to ANOVA analysis. WS—water stress condition; WW—well-watered condition.

Inoculant Treatments	Shoot N Concentrations (%)		Shoot Atom% ^15^N Excess (%)		% Ndfa		Total N Fixed (g/Plant)	
	WS	WW	WS	WW	WS	WW	WS	WW
1568/pVA3	2.23 ± 0.06 b	2.44 ± 0.07 b	3.98 ± 0.12 b	3.73 ± 0.16 b	18.9 ± 1.2 b	28.4 ± 0.9 b	0.13 ± 0.01 b	0.39 ± 0.04 b
A1501	2.17 ± 0.03 ab	2.38 ± 0.05 ab	4.17 ± 0.20 b	3.99 ± 0.22 b	15.1 ± 0.7 a	23.3 ± 1.0 a	0.09 ± 0.01 a	0.29 ± 0.02 a
*nifH*-mutant	2.18 ± 0.02 ab	2.30 ± 0.06 a	4.91 ± 0.13 a	5.21 ± 0.10 a	-	-	-	-
No inoculation	2.12 ± 0.08 a	2.31 ± 0.02 a	5.10 ± 0.09 a	5.34 ± 0.17 a	-	-	-	-

**Table 4:** Contents of soil ammonium, nitrate and organic C in the rhizospheric soil at day 50 of the maize growing season. The values are mean ± standard deviations (n = 16). Different letters (a–b) indicate a significant difference (*p* < 0.05) according to ANOVA analysis. WS—water stress condition; WW—well-watered condition.

Water Treatment	Inoculant Treatment	NH_4_^+^-N (mg/kg)	NO_3_^−^N (mg/kg)	Organic C (mg/kg)
WS	1568/pVA3	10.7 ± 1.03 b	5.42 ± 0.40 b	10.43 ± 0.05 a
	A1501	2.47 ± 0.11 a	3.91 ± 0.23 a	11.10 ± 0.03 a
	*nifH*-mutant	2.61 ± 0.06 a	4.04 ± 0.13 a	10.56 ± 0.07 a
	Non-inoculation	2.50 ± 0.09 a	3.90 ± 0.14 a	11.02 ± 0.02 a
WW	1568/pVA3	13.3 ± 1.26 b	8.91 ± 0.51 b	11.21 ± 0.10 a
	A1501	2.83 ± 0.07 a	6.23 ± 0.62 a	10.49 ± 0.06 a
	*nifH*-mutant	2.87 ± 0.10 a	6.42 ± 0.98 a	10.84 ± 1.02 a
	Non-inoculation	2.90 ± 0.08 a	6.73 ± 0.80 a	10.88 ± 0.09 a

**Table 5:** Shoot height (SH), shoot dry weight (SDW), shoot N concentrations (NC), length (LL) and width (WL) of leaf, diameter of shoot (DS), the row numbers per ear (RN) and grain numbers per row (GN), thousand seed weight (TSW) and grain yield (GY) of maize plants from the field experiment. 0 N, 0 kg of N ha^−1^; 75% N, 75 kg of N ha^−1^; 100% N, 100 kg of N ha^−1^. The values are mean ± standard deviations (n = 20). Different letters (a–f) indicate a significant difference (*p* < 0.05) according to ANOVA analysis.

Inoculant Treatments	Nitrogen Application	SH (cm)	SDW (g/Plant)	NC (mg/g)	LL (cm)	WL (cm)	DS (cm)	RN (no./Plant)	GN (no./Plant)	TSW (g)	GY (t/ha)
	0 N	245.1 ± 2.0 ab	232.1 ± 3.8 b	18.1 ± 0.2 b	97.9 ± 1.1 a	9.7 ± 0.1 b	2.76 ± 0.05 bc	16.6 ± 0.2 ab	37.1 ± 1.2 ab	369.4 ± 10.0 b	4.75 ± 0.04 b
1568/pVA3	75% N	250.2 ± 1.4 c	248.2 ± 1.3 c	19.3 ± 0.2 c	101.4 ± 1.5 b	10.3 ± 0.2 b	2.89 ± 0.07 c	16.8 ± 0.3 b	38.2 ± 1.0 b	416.3 ± 7.5 cd	10.65 ± 0.04 d
	100% N	253.2 ± 1.8 d	276.1 ± 4.2 f	21.3 ± 0.3 d	105.0 ± 1.0 c	11.0 ± 0.1 c	3.01 ± 0.08 d	16.9 ± 0.1 b	39.7 ± 1.3 bc	443.7 ± 8.8 e	12.15 ± 0.07 e
	0 N	242.8 ± 1.1 a	230.0 ± 1.7 b	17.4 ± 0.1 ab	97.0 ± 1.5 a	9.4 ± 0.1 a	2.69 ± 0.04 b	16.3 ± 0.2 a	35.7 ± 0.9 a	352.9 ± 9.1 a	4.55 ± 0.03 a
A1501	75% N	247.5 ± 2.9 bc	251.6 ± 2.3 c	18.9 ± 0.3 c	99.5 ± 1.7 b	9.9 ± 0.2 b	2.80 ± 0.06 c	16.4 ± 0.1 a	37.8 ± 1.8 b	402.5 ± 11.3 c	10.15 ± 0.08 c
	100% N	251.4 ± 1.0 cd	278.3 ± 4.5 f	19.9 ± 0.2 d	104.6 ± 1.4 c	10.9 ± 0.1 c	2.93 ± 0.04 d	16.8 ± 0.2 b	38.9 ± 1.6 bc	431.7 ± 9.2 e	11.60 ± 0.09 e
	0 N	241.2 ± 1.2 a	224.5 ± 1.6 a	17.2 ± 0.1 a	95.7 ± 1.2 a	9.2 ± 0.1 a	2.60 ± 0.07 a	16.2 ± 0.1 a	35.9 ± 1.5 a	350.6 ± 12.0 a	4.45 ± 0.05 a
*nifH*-mutant	75% N	249.9 ± 1.1 c	259.1 ± 1.9 d	18.8 ± 0.3 c	98.3 ± 1.4 ab	9.8 ± 0.1 b	2.86 ± 0.02 c	16.5 ± 0.1 a	38.2 ± 2.0 bc	402.5 ± 10.7 c	9.95 ± 0.08 c
	100% N	255.6 ± 2.7 d	270.4 ± 3.2 e	20.5 ± 0.2 d	104.2 ± 1.0 c	10.8 ± 0.2 c	2.98 ± 0.04 d	16.8 ± 0.3 b	39.9 ± 1.1 bc	434.7 ± 8.7 e	12.00 ± 0.04 e
	0 N	242.6 ± 2.8 a	228.5 ± 2.4 ab	17.5 ± 0.1 a	96.8 ± 1.0 a	9.3 ± 0.1 a	2.62 ± 0.03 a	16.3 ± 0.1 a	36.6 ± 1.3 a	355.3 ± 11.2 a	4.50 ± 0.05 a
No inoculation	75% N	249.1 ± 2.5 c	258.1 ± 2.9 d	19.0 ± 0.2 c	99.3 ± 1.3 ab	10.1 ± 0.1 b	2.83 ± 0.05 c	16.8 ± 0.3 b	38.9 ± 1.8 bc	410.1 ± 9.8 cd	10.05 ± 0.06 c
	100% N	254.4 ± 3.1 d	279.8 ± 3.1 f	20.7 ± 0.4 d	103.1 ± 2.2 bc	11.2 ± 0.1 c	2.99 ± 0.02 d	16.9 ± 0.2 b	40.2 ± 2.1 c	440.1 ± 10.5 e	11.90 ± 0.08 e

## Data Availability

Not applicable.
